# PseUpred-ELPSO Is an Ensemble Learning Predictor with Particle Swarm Optimizer for Improving the Prediction of RNA Pseudouridine Sites

**DOI:** 10.3390/biology13040248

**Published:** 2024-04-08

**Authors:** Xiao Wang, Pengfei Li, Rong Wang, Xu Gao

**Affiliations:** 1 School of Computer Science and Technology, Zhengzhou University of Light Industry, No. 136, Science Avenue, Zhengzhou 450002, China; wangxiao@zzuli.edu.cn (X.W.); 332107040630@email.zzuli.edu.cn (P.L.); 2 Henan Provincial Key Laboratory of Data Intelligence for Food Safety, Zhengzhou University of Light Industry, No. 136, Science Avenue, Zhengzhou 450002, China; 3 School of Electronic Information, Zhengzhou University of Light Industry, No. 136, Science Avenue, Zhengzhou 450002, China; wangrong@zzuli.edu.cn; 4 National Supercomputing Center in Zhengzhou, School of Computer and Artificial Intelligence, Zhengzhou University, Zhengzhou 450001, China

**Keywords:** pseudouridine, machine learning, RNA profile, One-Hot Encoding, K-mer, PSO

## Abstract

**Simple Summary:**

RNA pseudouridine modifications are present in various RNAs across different organisms and play crucial roles in regulating gene expression during biological processes. The accurate identification of pseudouridine sites within RNA sequences is essential for understanding their functional mechanisms. This study proposes a novel ensemble learning predictor named PseUpred-ELPSO, which accurately predicts RNA pseudouridine sites. The predictor demonstrates excellent performance in both cross-validation and independent testing. A user-friendly web server has been established, making it a powerful tool for pseudouridine site identification.

**Abstract:**

RNA pseudouridine modification exists in different RNA types of many species, and it has a significant role in regulating the expression of biological processes. To understand the functional mechanisms for RNA pseudouridine sites, the accurate identification of pseudouridine sites in RNA sequences is essential. Although several fast and inexpensive computational methods have been proposed, the challenge of improving recognition accuracy and generalization still exists. This study proposed a novel ensemble predictor called PseUpred-ELPSO for improved RNA pseudouridine site prediction. After analyzing the nucleotide composition preferences between RNA pseudouridine site sequences, two feature representations were determined and fed into the stacking ensemble framework. Then, using five tree-based machine learning classifiers as base classifiers, 30-dimensional RNA profiles are constructed to represent RNA sequences, and using the PSO algorithm, the weights of the RNA profiles were searched to further enhance the representation. A logistic regression classifier was used as a meta-classifier to complete the final predictions. Compared to the most advanced predictors, the performance of PseUpred-ELPSO is superior in both cross-validation and the independent test. Based on the PseUpred-ELPSO predictor, a free and easy-to-operate web server has been established, which will be a powerful tool for pseudouridine site identification.

## 1. Introduction

RNA pseudouridine is considered to the most abundant RNA modification site known, and it is also considered to be one of the most essential RNA modification sites found in both prokaryotes and eukaryotes [1]. Further, it is widely present in multiple classes of RNAs from several species, such as tRNA, mRNA, snRNA, snoR-NA, and rRNA [2]. For example, in tRNA, pseudouridine promotes its common structural motifs, and it is essential for ribosome assembly [3]. Additionally, in mRNA [4], pseudouridine can improve its translation efficiency, reduce the innate immune response caused by RNA, and so on. It is clear that the study of information on pseudouridine modification sites has implications for further unravelling the nature of the relevant biological links. However, genome-wide analysis experiments are expensive and time-consuming [5,6,7,8]. Facing the explosive growth of data in the post-genomic era, it is vital to develop computational methods that can rapidly and accurately identify pseudouridine sites in a timely manner. In recent years, several fast and inexpensive methods for identifying RNA pseudouridine sites have also emerged [9,10,11,12,13,14,15].

For example, Li et al. developed the first computational model called PPUS to predict PUS-specific pseudouridine sites in *Homo sapiens* and *Saccharomyces cerevisiae* [9]. Later, Chen et al. developed an iRNA-PseU predictor using the chemical properties of nucleotides and pseudo-nucleotide composition [10]. Inspired by these works, He et al. proposed PseUI using five different feature representations to further improve the accuracy of identifying RNA pseudouridine sites [11]. Later, Tahir et al. used convolutional neural networks to design a new predictor, iPseU-CNN, for identifying pseudouridine sites [12]. Liu et al. used the extreme gradient boosting algorithm to identify RNA pseudouridine sites with a predictor named XG-PseU [13]. Lv et al. developed a new predictor, RF-PseU, by fusing six feature representations based on the random forest algorithm [15], which achieved advanced results. However, existing works have relatively weak feature representation capabilities because they do not consider the optimal fusion of different feature representations. There is still room for improving the performance of these classifiers. 

Therefore, an innovative ensemble learning predictor PseUpred-ELPSO is proposed. It predicts RNA pseudouridine sites in *H. sapiens*, *S. cerevisiae*, and *M. musculus* datasets with good predictive accuracy. The idea of the PseUpred-ELPSO predictor is constructed using a stacking strategy combined with particle swarm optimization (PSO). Stacking strategy which is an ensemble strategy that combines multiple base classifiers via a meta-classifier, and the PSO is used to search the weight of the base classifiers. An efficient and easy-to-operate web server based on the PseUpred-ELPSO predictor has been established and can be accessed at http://www.xwanglab.com/PseUpred-ELPSO/ (accessed on 2 March 2024).

## 2. Materials and Methods

### 2.1. Benchmark Datasets

The three commonly used benchmark datasets and two independent test datasets, which came from iRNA-PseU [10], are employed in this study. The datasets used in the study are derived from RMBase [16], which is a database integrating over 100 types of RNA modifications, proposed by Sun et al. in 2015. The RMBase database is distributed under the terms of the Creative Commons Attribution License (http://creativecommons.org/licenses/by/4.0/ (accessed on 2 March 2024)), which permits unrestricted reuse, distribution, and reproduction in any medium, provided the original work is properly cited. With technological advancements, a new and more powerful version, RMBase v3.0 [17], has also been developed, which mainly focuses on the mechanism and function of diverse RNA modifications. It ensures a more comprehensive and consistent comparison. The three benchmark datasets contain an equal number of pseudouridine sequences and non-pseudouridine sequences, with 628 RNA sequences from *S. cerevisiae*, 990 from *H. sapiens*, and 944 from *M. musculus* species, respectively. There are 31 nucleotides in the RNA sequences from the *S. cerevisiae* dataset and 21 nucleotides in both the *H. sapiens* and *M. musculus* datasets. Finally, two independent datasets from *S. cerevisiae* and *H. sapiens* species are constructed, both containing 100 pseudouridine site sequences and 100 non-pseudouridine site sequences. The benchmark datasets and independent test datasets are available for download via the web server http://www.xwanglab.com/PseUpred-ELPSO/ (accessed on 2 March 2024).

### 2.2. Overview of PseUpred-ELPSO

**Figure 1:**
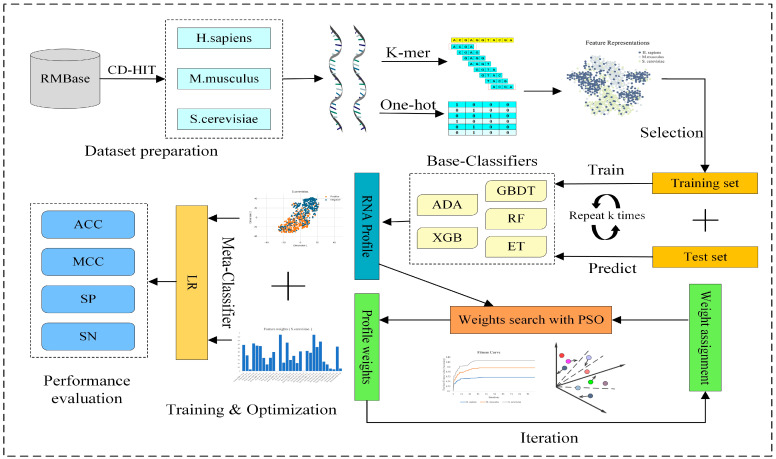
The overall framework of RNA pseudouridine site predictor PseUpred-ELPSO. Firstly, three benchmark datasets for three species (*H. sapiens*, *M. musculus*, and *S. cerevisiae*) are constructed. Secondly, six feature representations are constructed using One-Hot Encoding (k = 1, 2, and 3) and K-mer (k = 1, 2, and 3). Subsequently, 5 machine learning algorithms and 6 feature representations are utilized to construct 30 base classifiers, with each classifier built using optimal features obtained through a two-step feature selection strategy. Then, for the combination of these base classifiers, the predictions of these base classifiers are regarded as a new feature representation for RNA sequences, called the RNA profile. Then, PSO is used to search the optimal weights of the RNA profile to further enhance the representation ability of the RNA profile. Finally, a logistic regression classifier is employed as the meta-classifier to build the final predictor for pseudouridine site identification. ADA: adaptive boosting classifier; GBDT: gradient boosting decision tree classifier; XGB: extreme gradient boosting classifier; RF: random forest classifier; ET: extra trees classifier; PSO: particle swarm optimization; LR: logistic regression classifier; ACC: accuracy; MCC: Matthew’s correlation coefficient; SN: sensitivity; SP: specificity.

In this study, we propose a new meta-learning prediction method named PseUpred-ELPSO for the identification of RNA pseudouridine sites. The predictor PseUpred-ELPSO uses a stacking-based ensemble strategy combined with particle swarm optimization (PSO). The complete design and performance evaluation process of PseUpred-ELPSO is shown in detail in Figure 1. Firstly, this study argues that it would be efficient to use One-Hot Encoding for the RNA sequences by analyzing the nucleotide composition preferences between the RNA pseudouridine site sequences of the three species datasets. To complement the One-Hot Encoding feature representation, we also use a K-mer nucleotide frequency feature representation. Therefore, six feature representations are finally constructed, including One-Hot Encoding (k = 1, 2, and 3) and K-mer (k = 1, 2, and 3). Secondly, 5 machine learning algorithms and 6 feature representations are utilized to construct 30 base classifiers (6 feature representations × 5 machine learning classifiers), with each classifier built using optimal features obtained through a two-step feature selection strategy. The five machine learning algorithms are adaptive boosting (ADA) [18], gradient boosting decision tree (GBDT) [19], extreme gradient boosting (XGB) [20], random forest (RF) [21], and extra trees (ET) [22]. For the combination of these base classifiers, the predictions of the base classifiers are regarded as a new feature representation for RNA sequences, called the RNA profile. Then, PSO is used to search the optimal weights of the RNA profile to further enhance the representation ability of the RNA profile. The base classifiers’ outcomes, derived from K-fold cross-validation, serve as features. These features are then multiplied by the weights determined through particle swarm optimization (PSO), and the resultant values are subsequently input into the logistic regression (LR) model. Finally, the logistic regression (LR) classifier is selected as the meta-classifier for the construction of the pseudouridine identification predictor.

### 2.3. Feature Representation

#### 2.3.1. One-Hot Encoding

One-Hot Encoding is one of the most frequently used methods for sequence data preprocessing [23,24,25]. It is a representation of categorical variables as binary vectors, which in detail means that categorical values are mapped to integer values, each represented as a binary vector, with all values being 0 except for one valid value, which is encoded as 1. For example, in the RNA sequence, four nucleotides are marked using 0 and 1; thus, A, C, G, and U can be expressed as (1, 0, 0, 0), (0, 1, 0, 0), (0, 0, 1, 0), and (0, 0, 0, 1), respectively. Moreover, the K-nucleotides in RNA can be encoded as $4^{k}$(λ − k + 1)-dimensional vectors, where λ is the length of the RNA sequence. To obtain the most primitive and basic sequence information, we used One-Hot Encoding to directly represent the original RNA sequence. Meanwhile, the One-Hot Encoding of K-nucleotides with K = 1, 2, and 3 was performed, that is, the nucleotide, dinucleotide, and trinucleotide of the RNA sequence were encoded.

#### 2.3.2. K-mer Nucleotide Frequency (K-mer)

The K-mer nucleotide frequency (K-mer) feature counts the occurrence frequencies of k neighbor nucleotides and determines the mapping of a sequence to a digital space using a fixed-length digital feature vector. It is also one of the most widespread and effective feature descriptors for RNA sequence representation [26,27,28,29]. To effectively complement the feature representation encoded by One-Hot Encoding, in this study, K-mer was used to express RNA sequences. We also chose K = 1, 2, and 3, that is, the frequencies of the nucleotide, dinucleotide, and trinucleotide of the RNA sequence were counted.

### 2.4. Machine Learning

#### 2.4.1. Adaptive Boosting (ADA)

The ADA classifier [18] is an ensemble learning method used for solving classification problems. Its full name is the adaptive boosting classifier. The algorithm sequentially trains multiple weak learners (such as decision trees), weighing the data samples at each round of training to make the weak learners focus more on previously misclassified samples. Eventually, the predictions of all weak learners are combined with the weights to form the final classification result. The advantages of the ADA classifier include simplicity, efficiency, and resistance to overfitting.

#### 2.4.2. Gradient Boosting Decision Tree (GBDT)

The gradient boosting decision tree (GBDT) classifier [19] is a popular ensemble learning method used for classification tasks. It sequentially builds an ensemble of decision trees, where each tree is trained to correct the errors made by the previous trees. Unlike AdaBoost, which adjusts sample weights, the GBDT focuses on minimizing a loss function by optimizing the output of the ensemble. It is known for its robustness to overfitting and its ability to handle heterogeneous features and complex interactions in the data.

#### 2.4.3. Extreme Gradient Boosting (XGB)

The extreme Gradient Boosting (XGB) classifier [20] is based on the gradient boosting framework. XGBoost is an efficient machine learning algorithm, particularly suitable for classification and regression tasks. It achieves predictions by constructing multiple decision trees and combining them into a powerful ensemble model. XGBoost has many advantages, including highly optimized performance, flexible parameter tuning, and friendly handling of missing values. Due to its efficiency and accuracy in large-scale datasets, XGBoost has become one of the preferred models in many data science competitions and practical applications.

#### 2.4.4. Random Forest (RF)

The random forest (RF) classifier [21] is an integrated learning algorithm based on a decision tree. It can obtain accurate and stable predictions by building multiple decision trees and merging them. RF introduces randomness into the construction of each decision tree, including the random selection of samples and features, to improve the model’s generalization ability. RF classifiers typically exhibit good performance and robustness and are suitable for various classification tasks. RF is one of the commonly used algorithms in bioinformatics.

#### 2.4.5. Extra Trees (ET)

The extra trees (ET) classifier [22] is a type of ensemble learning method used for classification tasks. Similar to random forest, it also builds multiple decision trees, but with some key differences. In extra trees, each decision tree is constructed using a random subset of features and thresholds, without any pruning. Additionally, the final prediction is made by averaging the predictions of all trees in the ensemble. Extra trees is known for its efficiency and ability to handle high-dimensional datasets. It often exhibits competitive performance compared to other ensemble methods while requiring less computational resources.

### 2.5. Stacking Strategy Combined with Particle Swarm Optimization (PSO)

#### 2.5.1. Stacking Strategy

Stacking strategy is an ensemble technique that combines multiple classification models via a meta-classifier [30,31,32,33]. It contains two major steps, with the classifier for each step corresponding to the base classifier and the meta-classifier respectively. The base classifiers are trained first on the full training data, then all base classifier outputs are used as input features for the meta-classifier training. Firstly, the training data are split into k-folds, similar to k-fold cross-validation. Secondly, a base classifier is fitted to the k-1 parts and predictions are made for k-th part and test data. After that, each part of the training data will receive the predictions of the training data and k-times test data. Finally, predictions from the training data are used as training features for the meta-classifier, and the average of the predictions from the k-times test data are used as the testing features for the meta-classifier.

To make RNA sequences with a stronger representation, we utilize five stronger machine learning classifiers with tree-based ensembles as the base classifiers in the first step, including random forest (RF) [21], extra trees (ET) [22], adaptive boosting (ADA) [18], extreme gradient boosting (XGB) [20], and gradient boosting decision tree (GBDT) [19]. Then, to improve the robustness of the meta-classifier, a simpler logistic regression (LR) classifier is utilized as a meta-classifier in the second step. The implementations of these classifiers in this study are achieved by using the Scikit-Learn package [34].

#### 2.5.2. Feature Selection

We firstly pretrain the feature vectors using their respective corresponding base classifier to access the weights of the feature dimensions. All the calculated feature weights are then sorted from smallest to largest. Afterwards, the non-repeating feature weights are used as thresholds for feature selection, and feature filtering was performed to define the best RNA sequence information for each base classifier. Moreover, the optimized RNA feature representations are trained and the prediction results are used as the RNA profile. Hence, each RNA sequence is represented as a vector of 30 dimensions (5 base classifiers × 6 feature representations), which is the RNA profile.

#### 2.5.3. Particle Swarm Optimization (PSO)

Particle swarm optimization (PSO) is a frequently used swarm intelligence algorithm derived from the study of bird predation and designed by simulating the predatory behavior of birds. It can be regarded as a population-based optimization tool, and in an n-dimensional search space, individual (particle) potential problem solutions move to find the global optimal solution. Each particle records its previous optimal position in pbest vector and the global optimal position of the entire population in gbest vector. Information about the search space is shared by all particles. Therefore, each particle moves in the direction of its previous optimal position (pbest) and the global optimal position (gbest) in each iteration. In each iteration, the particle first calculates the velocity vector *v*, then it determines the direction of its motion and updates its position *x*. Assuming *t* is the number of iterations, the position of the (*t* + 1)-th iteration is the sum of the previous position $x_{i}\left(t\right)$ and its current velocity $v_{i}(t+1)$. Moreover, the it’s optimization logic searches for optimal solutions and all position vectors are assessed by the fitness function. More detailed information on the PSO algorithm is available in [35,36,37].

#### 2.5.4. PSO to Optimize the Weights of RNA Profile

PseUpred-ELPSO is a meta-learning framework that identifies RNA pseudouridine sites with good predictive accuracy. The core idea is to use a stacking strategy, which is an ensemble method that combines multiple base classifiers via a meta-classifier. In addition, this study also combines PSO to optimize the weights of the prediction of base classifiers, and the predictions from the outputs of the base classifiers are called the RNA profile.

In this study, the dimension of particle positions corresponds to the dimensionality of features, and the positions of particles represent the weights of features. During the optimization process, these particles continuously move to find the optimal solution. The criterion for evaluating the optimal solution is to take the positions (weights) and multiply them by the feature inputs to the logistic regression (LR) model to obtain the accuracy value. Through iterative refinement, the process can converge to approximate optimal feature weights. The particle population chosen contains 60 particles and 100 iterations to ensure sufficient population diversity and to guarantee sufficient global search. The position of each particle represents the weight of each RNA profile, taking values in the range of [0, 1], with the inertia weight set to 0.8, and both the personal learning factor c_1_ and the social learning factor c_2_ set to 0.6. Finally, to obtain more effective weights, the accuracy of 10-fold cross-validation for meta-classifiers is used as the fitness value. This not only ensures that the population of particles moves towards the direction of high classification accuracy, but also prevents overfitting.

### 2.6. Model Evaluation

Four metrics for cross-validating computational models are commonly used in the field of bioinformatics to assess the quality of models. There is sensitivity (SN), accuracy (ACC), specificity (SP), and Matthew’s correlation coefficient (MCC). Their formulas are as follows:

(1) $$SN=\frac{TP}{TP+FN}$$

(2) $$ACC=\frac{TP+TN}{TP+TN+FN+FP}$$

(3) $$SP=\frac{TN}{TN+FP}$$

(4) $$MCC=\frac{TP\times TN-FP\times FN}{\sqrt{\left(TP+FN)(TP+FP)(TN+FP)(TN+FN\right)}}$$

TP (true positive) means that both the observation and prediction are positive, namely, it is the number of true PseU sites correctly predicted; FN (false negative) means that the observation is positive but the prediction is negative, namely, it is the number of true PseU sites predicted to be non-PseU sites; TN (true negative) means that both the observation and prediction are negative, namely, it is the number of non-PseU sites correctly predicted; FP (false positive) means that the observation is negative but the prediction is positive, namely, it is the number of non-PseU sites predicted to be true PseU sites.

Therefore, SN is the probability of obtaining the correct prediction of PseU sites. SP is the probability of obtaining the correct prediction of non-PseU sites. ACC represents the prediction accuracy of the entire site. Since the MCC considers the true positive, true negative, false positive, and false negative, it is generally regarded as a measure of balance.

## 3. Results

### 3.1. Nucleotide Composition Preference Analysis

**Figure 2:**
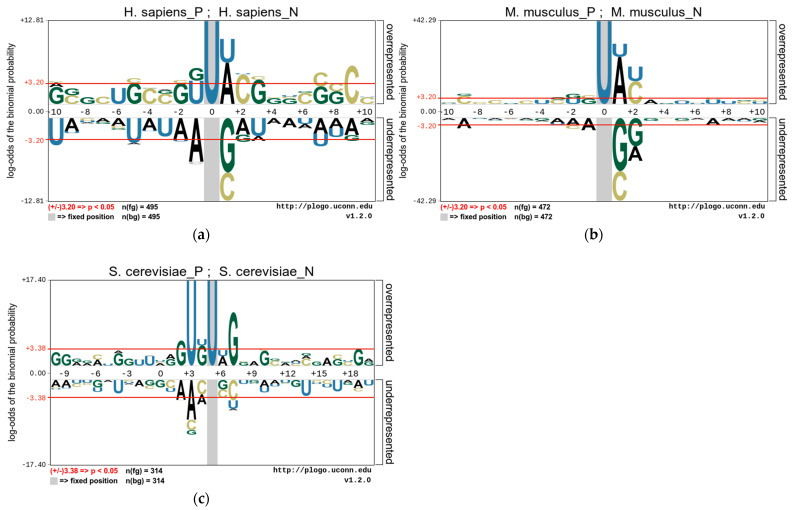
The nucleotide preferences of RNA sequences in three benchmark datasets (**a**). *H. sapiens*, (**b**) *M. musculus*, and (**c**) *S. cerevisiae*.

In this study, we use the pLogo tools [38] to analyze the position-specific preferences of the nucleotide composition of these species and to explore the statistically significant nucleotide differences between sequences containing PseU sites and non-PseU sites. Therefore, we find that there is an obvious preference between RNA sequences containing PseU sites and non-PseU sites in the datasets of the three species. Figure 2 shows the nucleotide preferences of 21 base pairs of RNA sequences from the *M. musculus* and *H. sapiens* datasets, and the nucleotide preferences of 31 base pairs of RNA sequences from the *S. cerevisiae* dataset, where the U base pair is centrally positioned. As shown in Figure 2, the nucleotide distributions of each species are significantly different. A comparison of 495 PseU and 495 non-PseU sample sequences of *H. sapiens*, for example, reveals that adenosine (A) and uridine (U) at position +1 are significantly enriched in sequences containing PseU sites, while sequences at non-PseU sites exhibit significant cytosine (C) and guanine (G) preferences at position +1, and cytosine (C) at position +9 and uridine (U) at positions −5 and −9 are significantly enriched. The positions of nucleotide enrichment in *M. musculus* are similar to those in *H. sapiens* apart from G and U, which are enriched at positions −1 and −2. In *S. cerevisiae*, U and G are significantly enriched in upstream and G and C are significantly enriched in downstream regions on sequences containing PseU sites, with A, C, and U also enriched in different positions on sequences containing non-PseU sites.

The above results demonstrate that the position of a nucleotide in a sequence is a key factor in the outcome of the distinction between PseU and non-PseU sites, and it is reasonable to use sequence information to establish a computational approach for identifying PseU sites. The One-Hot Encoding used in this study is a direct coding of the original RNA sequences and it represents the most original RNA sequence information preferences which have significant contribution in distinguishing PseU sites from non-PseU sites. Therefore, if the preference information is fully used, then it is very beneficial to improve the identification accuracy of PseU sites. In addition, the statistical frequencies of nucleotide, dinucleotide, or trinucleotide occurrences in sequences are a complement to this original sequence information.

### 3.2. RNA Profile Analysis

**Figure 3:**
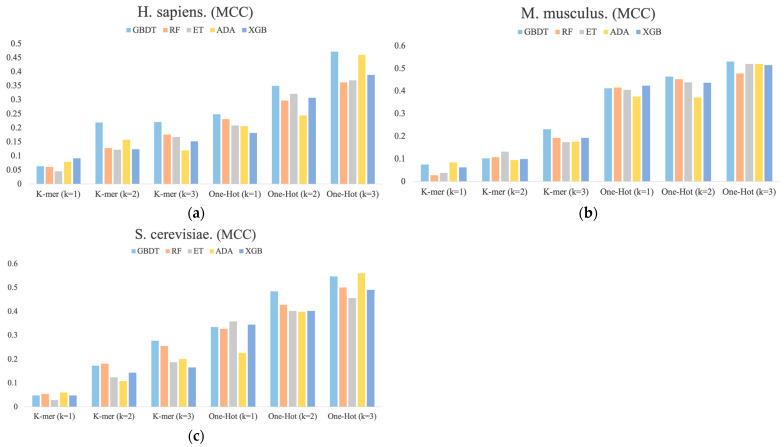
The MCC values for each dimension of RNA profiles obtained by the prediction of six feature representations. (**a**) *H. sapiens*, (**b**) *M. musculus*, and (**c**) *S. cerevisiae*.

Figure 3 shows the MCC values determined for each dimension of the RNA profile in the *M. musculus*, *H. sapiens*, and *S. cerevisiae* species datasets. It can be found that the performance of different base classifiers on different feature representations is not uniform. For example, for *H. sapiens*, the One-Hot Encoding (k = 3) feature representation performs better than the other four base classifiers for the ADA classifier, but it has the lowest performance for One-Hot Encoding (k = 1) and K-mer (k = 2). For *S. cerevisiae*, the ET classifier is inferior to the other four base classifiers in most feature representations, but it outperforms them all in One-Hot Encoding (k = 1) feature representation. In addition, the performance of these feature representations is also inconsistent, with performance increasing sequentially across the three species datasets and the latter outperforming the former overall. Thus, these differences between the performances of different classifiers with different features are necessary for integration.

**Figure 4:**
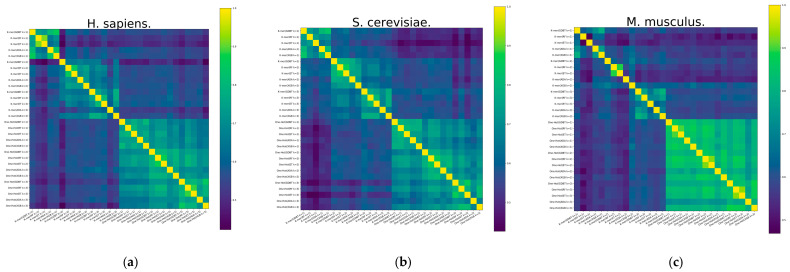
The heatmap of Pearson correlation coefficients between different RNA profiles of datasets. (**a**) *H. sapiens*, (**b**) *S. cerevisiae*, and (**c**) *M. musculus*.

To expose the reasons for the improved model performance of the ensemble approach, a diversity analysis of the 30 base classifiers is carried out using the Pearson correlation coefficient method. Pearson correlation coefficients between RNA profiles are calculated and shown in Figure 4. It demonstrates the heatmap of the *S. cerevisiae* and *H. sapiens* species datasets, and it can be found that the Pearson correlation coefficient has similarity between different base classifiers with the same feature representation. It also illustrates the differences between the RNA profiles of the *M. musculus* species dataset. However, there is significant heterogeneity between different features, especially between the two feature representations of K-mer and One-Hot Encoding, which can also indicate that K-mer is a valid complement to One-Hot Encoding features.

### 3.3. The Stacking Strategy Combined with PSO Improved the Performance

**Figure 5:**
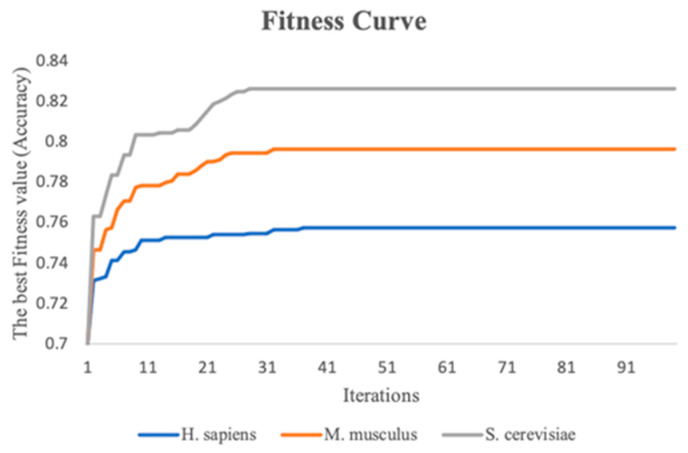
The fitness curves of the three benchmark datasets with iterations increasing.

This study performs the stacking strategy combined with PSO in three benchmark datasets. To visualize the experimental process more directly, fitness curves are plotted to show the change in global optimal accuracy over the course of the iterative 10-fold cross-validation process. Figure 5 indicates the fitness curves of the benchmark datasets. As the number of iterations increases, it is seen that the fitness curve of the three species datasets increases, and the accuracy of the 10-fold cross-validation of the meta-classifier also continuously improves. In addition, the 10-fold cross-validation accuracy of the meta-classifier for the *M. musculus*, *S. cerevisiae*, and *H. sapiens* species datasets improved by a total of 5.0%, 6.3%, and 2.6%, respectively, compared to the first iteration.

To validate the effectiveness of the stacking strategy combined with PSO proposed in this study, we compare it with the original stacking strategy. As can be seen in Table 1, the results of the experiments clearly show that the ensemble predictor has been further improved significantly in the three benchmark datasets. Compared to the original stacking strategy, the stacking strategy combined with PSO provides varying degrees of improvement in the four metrics. Especially for MCC, the stacking strategy combined with PSO improves over the range of 3.0–7.0% in the three species datasets. Additionally, the ACC values of the *S. cerevisiae*, *M. musculus*, and *H. sapiens* datasets have also been improved, with an increase of 3.1%, 2.1%, and 1.2%, respectively. In addition, the SN value of the *S. cerevisiae* dataset has increased by 4.0%, the SP value of the *M. musculus* dataset has improved by 2.4% and it exceeds 80%. These results show that the stacking strategy combined with PSO outperforms the original stacking strategy in the benchmark dataset; meanwhile, they also indicate that the RNA profile weights learned by PSO are effective.

**Figure 6:**
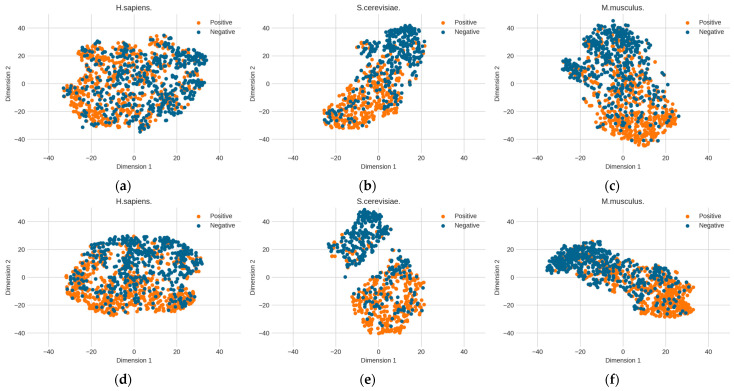
The changes in the distribution of the three benchmark datasets before and after determining the optimal weights by particle swarm optimization (PSO). (**a**) *H. sapiens* (before PSO), (**b**) *S. cerevisiae* (before PSO), (**c**) *M. musculus* (before PSO), (**d**) *H. sapiens* (after PSO), (**e**) *S. cerevisiae* (after PSO), and (**f**) *M. musculus* (after PSO).

The above optimized RNA profile weights are proven to be effective in improving performance. However, it is unknown how the learned features contribute to performance improvement. Here, we further explore the change in feature space from the original distribution to the optimized distribution. The T-distributed stochastic neighbor embedding (t-SNE) technology [39] was used to reduce the dimensionality of the feature space and to visualize the feature space. Figure 6 shows the changes in the distribution of the three benchmark datasets before and after determining the optimal weights. The diversities between the original RNA profile and the optimized RNA profile can be observed. Before the RNA profiles are optimized, the distribution of positive and negative samples is relatively dispersed. However, after optimization, the two clusters of positive and negative samples are obviously closer and the boundary is clearer than in the original RNA profile, which is particularly obvious in the *S. cerevisiae* dataset. This shows that determining the optimized genomic sequence information makes it easier to distinguish between positive samples (PseU sites) and negative samples (non-PseU sites).

**Figure 7:**
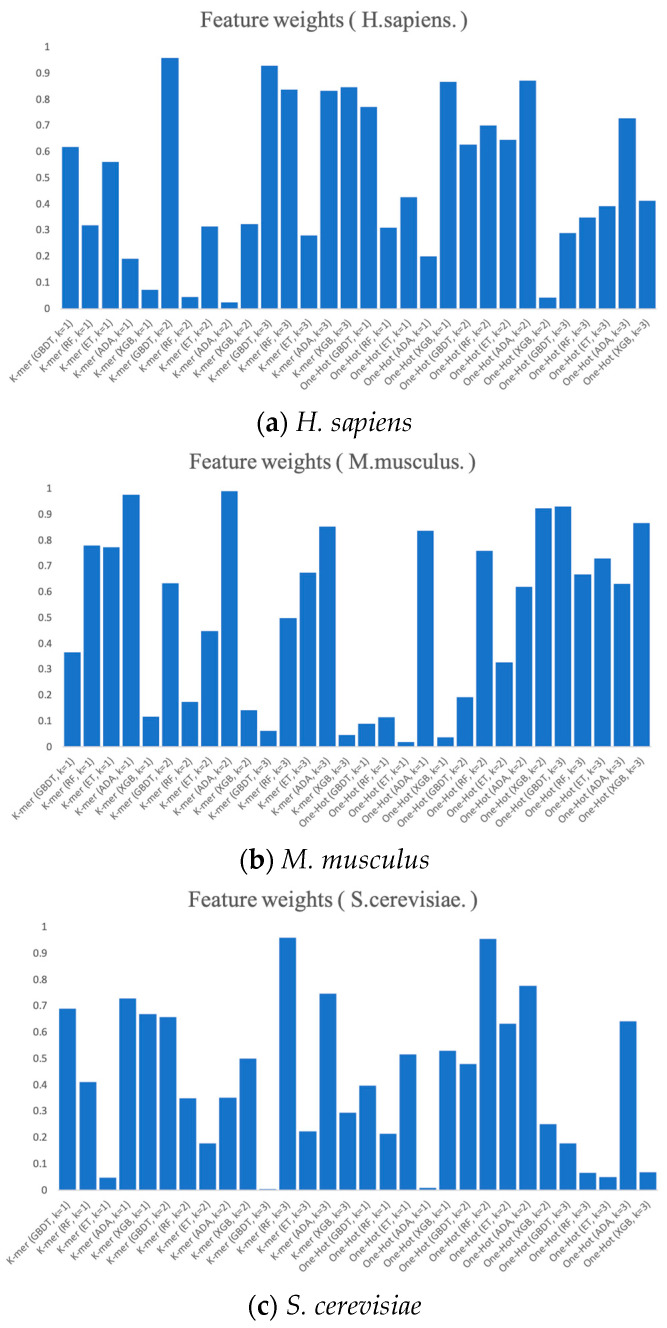
The RNA profile weights obtained for each dimension by the prediction and PSO search of six feature representations.

Finally, we present the RNA profile weights obtained in the three benchmark datasets using histograms, as shown in Figure 7. For example, in *H. sapiens*, the optimized RNA profile weights of PSO reaches the highest in K-mer (k = 2) when GBDT is the base classifier, with the weight value of almost 1. Meanwhile, the weights of K-mer (GDBT, k = 3), One-Hot (XGB, k = 1), and One-Hot (ADA, k = 2) all reach higher levels. Moreover, the weights of the base classifier of K-mer (ADA, k = 2) and One-Hot (XGB, k = 2) almost reach 0. Both the weights of K-mer (GBDT, k = 3) and One-Hot (ADA, k = 1) in *S. cerevisiae* and the weights of One-Hot (ET, k = 1) and One-Hot (XGB, k = 1) in *M. musculus* almost reach 0. The magnitude of these weights can be thought of as the degree of importance of integrating these base classifiers. The higher the degree of importance, the greater the weight assigned to it, which indicates that it plays a more significant role in the integration.

### 3.4. Comparison with State-of-the-Art Predictors

To prove the superiority of the PseUpred-ELPSO prediction for identifying PseU sites, the same benchmark training and independent test datasets used by several state-of-the-art methods are used in this study to critically evaluate and compare the predictive performance of PseUpred-ELPSO, including iRNA-PseU, RF-PseU, PseUI, iP-seU-CNN, XG-PseU, and iPseU-CNN. Table 2 illustrates the comparison between the most advanced predictor and PseUpred-ELPSO. In the *H. sapiens* and *S. cerevisiae* datasets, the accuracy of PseUpred-ELPSO is 74.8% and 82.6%, which is 7.8% and 11.4% higher than the last predictor (RF-PseU), respectively. Additionally, PseUpred-ELPSO’s MCC, SN, and SP has been increased by 23%, 11.4%, and 11.6% compared with the last predictor RF-PseU, respectively, in *H. sapiens*. As for the *M. musculus* dataset, compared with the current state-of-the-art predictors, the accuracy of the PseUpred-ELPSO predictor also exceeds the range of 4.9–10.6%. Generally, the 10-fold cross-validation result of the PseUpred-ELPSO predictor in the three benchmark datasets obtains better performance than other state-of-the-art predictors in the four evaluation indicators.

### 3.5. Comparative Analysis on Independent Datasets

This study validates the generalization ability of the model’s predictor for identifying pseudouridine sites in an independent dataset established by Chen et al. [8]. It contains two species datasets, *S. cerevisiae* and *H. sapiens*. While applying the predictor PseUpred-ELPSO to independent test datasets, four evaluation indicators were calculated, and the detailed comparative results can be obtained from Table 3. As can be seen in Table 3, compared with the other state-of-the-art predictors, the test results show that the PseUpred-ELPSO predictor outperforms them in the *H. sapiens* and *S. cerevisiae* datasets. Especially in the *S. cerevisiae* dataset, the MCC value of the independent test sets break through to the value of 60% for the first time, and the accuracy of the *S. cerevisiae* dataset also reaches 79.5%, which is increased by 2.5–19.5%. For the *H. sapiens* dataset, we also see a small enhancement. The ACC, MCC, and SP are 1%, 2%, and 3% higher than the last predictor RF-PseU, respectively. The averages of the ACC, MCC, SN, and SP for the PseUpred-ELPSO predictor reach 77.8%, 56%, 76.5%, and 80%, respectively. If viewed together, these results show that PseUpred-ELPSO achieves competitive performance compared to current state-of-the-art predictors and it does not suffer from overfitting.

## 4. Web Server for PseUpred-ELPSO

**Figure 8:**
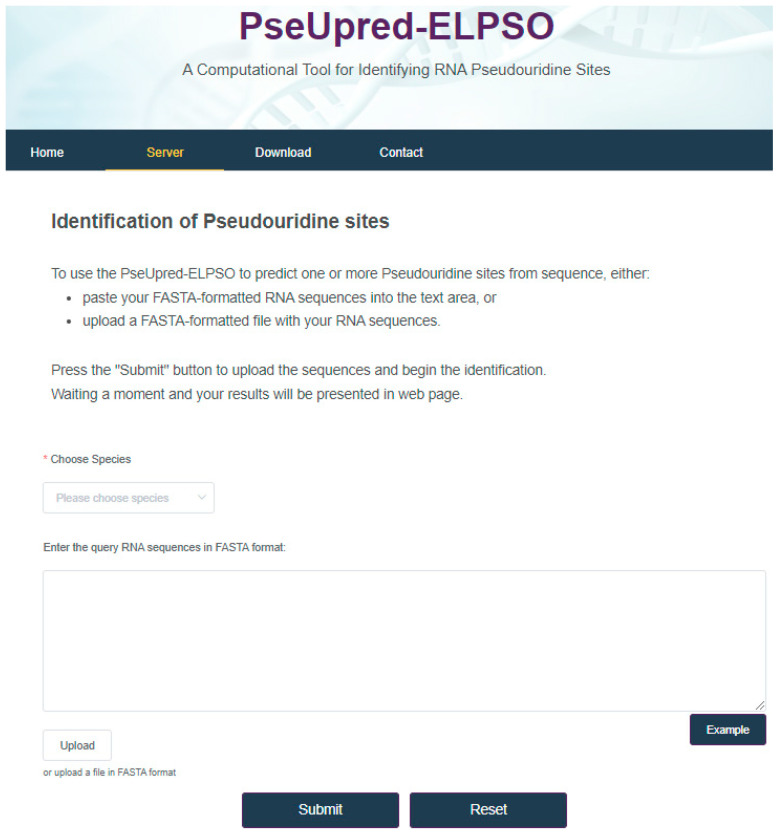
Web server for PseUpred-ELPSO.

In this work, for the convenience of most experimental scientists, the web server for PseUpred-ELPSO has been established at http://www.xwanglab.com/PseUpred-ELPSO/ (accessed on 2 March 2024) as shown in Figure 8. To use the PseUpred-ELPSO to predict pseudouridine sites from one or more RNA sequences, one can either paste their FASTA-formatted RNA sequences into the text area, or upload a FASTA-formatted file containing the RNA sequences, then press the “Submit” button to obtain the prediction results, waiting a moment before the prediction results are presented on a web page.

## 5. Discussion

In this study, an innovative PseUpred-ELPSO predictor, which predicts RNA pseudouridine sites with good predictive accuracy in *S. cerevisiae*, *M. musculus*, and *H. sapiens* datasets, is proposed, and the idea of the constructed PseUpred-ELPSO is to use a stacking strategy combined with particle swarm optimization. Firstly, we thought that it would be efficient to use One-Hot Encoding for the RNA sequences by analyzing the nucleotide composition preferences between the RNA pseudouridine site sequences of the three species datasets. Subsequently, to complement the One-Hot Encoding feature representation, we also used K-mer feature representation. Secondly, we utilized five machine learning classifiers with a tree-based ensemble as base classifiers, and the optimal features for each base classifier were filtered by a two-step feature selection strategy with an extensive performance comparison. To combine these multiple base classifiers, the predictions of the base classifiers were regarded as the RNA profile. Moreover, PSO was used to search the weight of the RNA profile to further enhance the representation of the RNA profile. Finally, the logistic regression (LR) classifier was used as a meta-classifier to construct the pseudouridine site identification predictor.

## 6. Conclusions

This study introduces an innovative predictor called PseUpred-ELPSO, which accurately predicts pseudouridine sites in RNA data sets from yeast (*S. cerevisiae*), mouse (*M. musculus*), and humans (*H. sapiens*). The construction of this predictor involves using One-Hot Encoding and K-mer feature representation for RNA sequences. It utilizes five tree-based ensemble machine learning classifiers, performs feature selection through a two-step strategy, combines the predictions of these base classifiers into an RNA profile, optimizes the RNA profile using particle swarm optimization (PSO), and finally employs a logistic regression (LR) classifier as a meta-classifier to build the pseudouridine site identification predictor. Compared to state-of-the-art predictors, PseUpred-ELPSO demonstrates superior performance in both cross-validation and independent tests, making it a promising tool for pseudouridine site identification.

## Figures and Tables

**Table 1:** 10-fold cross-validation scores of the original stacking and PSO-optimized stacking.

Species	Type	ACC	MCC	SN	SP
*H. sapiens*	Stack	0.745	0.49	0.758	0.734
	Stack (PSO)	0.757	0.52	0.775	0.742
*S. cerevisiae*	Stack	0.795	0.59	0.820	0.773
	Stack (PSO)	0.826	0.66	0.860	0.799
*M. musculus*	Stack	0.776	0.55	0.774	0.779
	Stack (PSO)	0.797	0.59	0.790	0.803

**Table 2:** Comparison of the 10-fold cross-validation scores of PseUpred-ELPSO with current state-of-the-art predictors.

Species	Predictor	ACC	MCC	SN	SP
*S. cerevisiae*	PseUI	0.641	0.29	0.647	0.643
	IRna-PseU	0.645	0.29	0.647	0.643
	XG-PseU	0.682	0.37	0.668	0.695
	iPseU-CNN	0.682	0.37	0.664	0.705
	RF-PseU	0.748	0.49	0.772	0.724
	PseUpred-ELPSO	0.826	0.66	0.860	0.799
*H. sapiens*	IRna-PseU	0.604	0.21	0.610	0.598
	PseUI	0.642	0.28	0.649	0.636
	RF-PseU	0.643	0.29	0.661	0.626
	XG-PseU	0.661	0.32	0.635	0.687
	iPseU-CNN	0.660	0.34	0.650	0.680
	PseUpred-ELPSO	0.757	0.52	0.775	0.742
*M. musculus*	IRna-PseU	0.691	0.38	0.733	0.648
	PseUI	0.704	0.41	0.799	0.703
	iPseU-CNN	0.718	0.44	0.748	0.691
	XG-PseU	0.720	0.45	0.765	0.676
	RF-PseU	0.748	0.50	0.731	0.765
	PseUpred-ELPSO	0.797	0.59	0.790	0.803

**Table 3:** Comparison of independent testing scores of PseUpred-ELPSO with current state-of-the-art predictors.

Species	Predictor	ACC	MCC	SN	SP
*H. sapiens*	IRna-PseU	0.65	0.30	0.600	0.700
	PseUI	0.655	0.31	0.630	0.680
	iPseU-CNN	0.690	0.40	0.777	0.608
	XG-PseU	0.675	/	/	/
	RF-PseU	0.750	0.50	0.780	0.720
	PseUpred-ELPSO	0.760	0.52	0.770	0.750
*S. cerevisiae*	IRna-PseU	0.600	0.20	0.630	0.570
	PseUI	0.685	0.37	0.650	0.720
	iPseU-CNN	0.735	0.47	0.686	0.778
	XG-PseU	0.710	/	/	/
	RF-PseU	0.770	0.54	0.750	0.790
	PseUpred-ELPSO	0.795	0.60	0.760	0.850

## Data Availability

The server and data for PseUpred-ELPSO are available at http://www.xwanglab.com/PseUpred-ELPSO/ (accessed on 2 March 2024).
